# Neuropsychology of Neuroendocrine Dysregulation after Traumatic Brain Injury

**DOI:** 10.3390/jcm4051051

**Published:** 2015-05-20

**Authors:** Josef Zihl, Osborne F. X. Almeida

**Affiliations:** 1 Department of Psychology, LMU University of Munich, 80802 Munich, Germany; 2 Max Planck Institute of Psychiatry, 80804 Munich, Germany; E-Mail: osa@mpipsykl.mpg.de

**Keywords:** traumatic brain injury, mental disorders, neuroendocrine dysregulation, cognition, mood

## Abstract

Endocrine dysfunction is a common effect of traumatic brain injury (TBI). In addition to affecting the regulation of important body functions, the disruption of endocrine physiology can significantly impair mental functions, such as attention, memory, executive function, and mood. This mini-review focuses on alterations in mental functioning that are associated with neuroendocrine disturbances in adults who suffered TBI. It summarizes the contribution of hormones to the regulation of mental functions, the consequences of TBI on mental health and neuroendocrine homeostasis, and the effects of hormone substitution on mental dysfunction caused by TBI. The available empirical evidence suggests that comprehensive assessment of mental functions should be standard in TBI subjects presenting with hormone deficiency and that hormone replacement therapy should be accompanied by pre- and post-assessments.

## 1. Introduction

TBI represents one of the leading causes of cognitive, emotional, and motor disability in both, industrialized and non-industrialized countries; falls, road, occupational and recreational accidents, and violence are the most frequent causes of TBI [1].

Functional impairments after brain injury are usually explained in terms of morphological and pathophysiological alterations of cortical and subcortical brain structures. This view is consistent with the fact that injury to local and distributed components of brain networks, and their interplay, can alter cognition, affect, and behavior [2]. While functional impairments after acquired brain injury are sequelae of grey (cortex) and white matter (subcortical fiber connections), endocrine hormones also play an essential role in maintaining brain function, including cognitive performance and the regulation of motivation/intention and emotion [3]. The most commonly reported cognitive deficits in moderate and severe TBI include impaired processing speed (“cognitive slowing”), which can affect practically all other cognitive domains, difficulties with concentration (elevated distractibility), and divided attention (decreased multi-tasking), memory, and executive function. Residual cognitive deficits may persist and may interfere with returning to or gaining employment and independent living. Reduced information processing speed and difficulties with concentration and divided attention are the most common deficits in mild TBI (for a review, see [4]). The risk for psychopathological symptomatology does not depend on TBI severity [5]; conditions such as major depression (~14%–42%), posttraumatic stress disorder (PTSD; ~14%), anxiety disorders (~9%), and bipolar disorders (~4%) can occur and persist after TBI [6]. Both, diffuse and focal lesions following TBI can explain cognitive and psychopathological alterations. Diffuse axonal injury may cause more global cognitive dysfunction, for example, reduced information processing and mental speed, sustained attention, concentration and divided attention, whereas focal injury (e.g., contusion) causes deficits that are more specific. Frontal and temporal contusions are common in TBI, and are responsible for memory and executive dysfunction, but they may also influence affect. Furthermore, vascular compromise and edema can disrupt blood supply and oxygen delivery to tissues, thus contributing to neuropsychological deficits; the latter vary according to the site of injury. Thus, TBI may be seen as a more “generalized” brain injury, causing nonspecific functional impairments, e.g., cognitive slowing; more specific functional deficits in the domains of attention, memory and executive function result from focal injury to grey and white matter contusion or traumatic complications [7,8].

This mini-review focuses on alterations in mental functioning (cognition, mood) that are associated with neuroendocrine disturbances in adults who have suffered traumatic brain injury (TBI); it also considers the role of hormones in brain plasticity. Specifically, the article will address the following issues: (a) hormonal contributions to the regulation of certain mental functions; (b) the consequences of TBI on mental health and neuroendocrine homeostasis; and (c) the effects of hormone replacement on mental dysfunction caused by TBI. Our review of the available empirical evidence suggests that improvements in the identification and management of hormone deficiencies in TBI subjects requires the inclusion of comprehensive, standardized neuropsychological assessment; this will help better understand and explain functional impairments in TBI.

## 2. Hormones and Mental Functions

Abundant evidence in humans and animals points to the important role of the endocrine system in brain development and the regulation of cognitive, emotional, affective, motivational, and social behaviors; endocrine hormones also regulate behaviors that are essential for individual and species survival (sleep, appetite, thirst, reproductive). Space restrictions allow us to illustrate this by just a few examples that represent the different classes of endocrine secretions.

A large array of peptides of cerebral origin (e.g., thyrotropin-releasing hormone, growth hormone-releasing hormone, corticotrophin-releasing hormone, arginine vasopressin, oxytocin), best known for their role in the regulation of the peripheral endocrine tissues, exert direct (intracerebral) or indirect effects on cognition and mood [3]. The pituitary polypeptide hormone growth hormone, better known as key regulator of somatic growth and metabolism, is essential for normal brain development as well as cognitive function such as memory [9,10,11]. Insulin, another polypeptide, appears to be essential for optimal functioning of various cortical and subcortical structures, in particular the hippocampus and prefrontal cortex. In light of the role of insulin in regulating blood glucose levels, it is interesting that, hyper- and hypoglycemic states are frequently accompanied by affective symptoms and impaired cognitive performance [12,13,14].

Low-molecular weight non*-*peptidergic hormones produced in the periphery also exert strong effects on brain functions. These hormones (e.g., gonadal steroids, such as estrogen and testosterone, adrenal steroids, such a cortisol) pass across the blood-brain barrier because of their relative lipophilicity or through binding to specific transporters (e.g., thyroid hormones). All of them can elicit long-lasting effects in the brain by binding to receptors that are ligand-activated transcription factors. Receptors for each of these classes of hormone are present in diverse brain regions and, are especially abundant in corticolimbic structures as well as the hypothalamus. Although primarily implicated in thermogenic processes, thyroid hormones (e.g., tri-iodothyronine and thyroxine) are crucial for the maturation of neural cells and are essential for learning and memory and overall cognitive performance [15,16]. Hyper- and hypothyroid states are associated with reduced executive function [17] and hyperthyroidism may cause irritability, restlessness, hyperexcitability, and emotional instability [18]. Sex steroids, in particular 17β-estradiol, play an important role in sexual differentiation of the brain and expression of sex-specific cognitive and affective behaviors [19]; they also enhance cognition, in particular memory [20]. Glucocorticoids (GC, e.g., cortisol), are released from the adrenal glands as part of the adaptive response to stress; as their name indicates, their main function is to regulate glucose and lipid metabolism [21]. In addition, glucocorticoids are potent suppressors of the immune response. Persistently high GC levels, such as those found under conditions of chronic stress, disrupt homeostasis (e.g., cardiovascular, metabolic) and may lead to the development of psychopathological symptoms such as depression [22]. In addition, GC influences cognition (e.g., attention, and visual and verbal memory) in a U-shaped dose-related manner [21,23]. It is, thus, not surprising that many subjects with major depression present with cognitive impairments. Neuropsychological studies revealed that hypercortisolism is more strongly associated with superordinate executive dysfunction rather than specific memory deficits [24]. This finding is in line with high expression levels of glucocorticoid receptors in the prefrontal cortex, hippocampus, amygdala, thalamus, and hypothalamus [25]. Thyroid hormones, and the above-mentioned sex and adrenal steroids all participate in the birth, differentiation and survival of neurons and/or glial cells. In addition, progesterone, a steroid produced by both the ovaries and brain, appears to influence neuronal repair and survival; the latter actions have been ascribed to progesterone derivatives (the so-called neurosteroids) that modulate the electrophysiological activity of neurons by binding to an allosteric binding site on the γ-aminobutyric acid (GABA) A receptor.

## 3. Neuroendocrine Dysregulation and Mental Disorders after TBI

Irrespective of severity of TBI, the vast majority of subjects report cognitive impairments and psychopathological symptoms, with attentional, memory, and executive deficits being the most frequent cognitive sequelae [26]. Chronic mental fatigue is a common disabling phenomenon in TBI subjects [27] and may reflect injury to the ventromedial prefrontal cortex [28]. Symptoms of major depression may be present in about a third of 91 subjects with TBI, of which about 75% exhibited comorbid anxiety and about 60% exhibited aggressive behavior [29]. Post-TBI patients also report reduced quality of sleep [30], which may contribute to their complaints of anxiety and depression.

Traumatic brain injury is often accompanied by chronic neuroendocrine dysfunction due to pituitary insufficiency or “hypopituitarism”. Schneider *et al.* [31,32] reported at least one pituitary function anomaly in 56% of 78 TBI subjects; in a systematic review of 19 studies (1137 cases), the same authors reported hypopituitarism in about 28% of TBI subjects Schneider *et al.* [33].

Reduced anterior pituitary activity is commonly associated by dysregulation of anterior pituitary-dependent growth, thyroid, and GC hormone secretion [34], as well as antidiuretic hormone (ADH) [35]. While ADH deficiency may contribute to diabetes insipidus in some patients [36], it may also underpin hyponatremia, a serious complication of up to 33% of subjects who suffered TBI [35,37].

Given that TBI patients may suffer from multiple endocrine deficiencies [38,39], it is difficult to attribute particular cognitive and affective symptoms to disruption of a single hormone. In this regard, it is worth noting that TBI subjects may present with disparate cognitive and affective symptoms that may appear as general impairments. For example, hypopituitarism after TBI may be associated with poor life quality and higher rates of depression and fatigue [40]. This is better illustrated by the results reported by Popovic *et al.* [41], who observed reduced visual and verbal memory and visuo-constructive abilities, impaired executive function, and mild-to-moderate depressive symptoms among TBI patients with hypopituitarism (34% of a total of 67 patients who had suffered TBI within the last 12 months). Pavlovic *et al.* [42] found no association between GH deficiency and chronic cognitive impairments more than one year after TBI. In contrast, other authors reported an association between growth hormone insufficiency and persistent (≥12 months) impairments in attention and verbal memory [43] and, irrespective of severity of TBI, reductions in energy and emotional well-being, as well as increased fatigue and depressive symptomatology in a smaller number (18% of 44 subjects; [44]). In addition, León-Carrión *et al.* [45] reported impaired attention, memory and executive function and affective symptoms in patients with severe TBI who developed GH deficiency.

The results of hypopituitarism on mental functions support the view that hormones play an important role for optimal functioning of brain systems involved in cognition and mood. Although cognition and mood were assessed directly in only a few studies, and despite the fact that dysfunctions differ with respect to frequencies as well as examined cognitive and affective domains, it is plausible to assume that all domains of cognition, *i.e.*, attention, memory, and executive function, can be impaired. It is possible that endocrine dysfunction mainly affects regulatory mental processes; this would explain why executive components of cognition are mainly impaired, with secondary consequences for information processing, attention and memory. Although no data are available yet on specific effects of hypocortisolism, hypothyroidism, and diabetes insipidus in subjects with TBI, it seems reasonable to expect that TBI patients and subjects with respective hormonal dysfunction without brain injury (see above) would show similar mental impairments. Evidence for this could be obtained by comparing mental functions in healthy subjects, and patients with hypopituitarism caused by TBI *vs.* all other causes.

Perusal of the literature indicates that TBI-associated hypopituitarism mainly affects executive functions, with lesser effects on information processing, attention and memory systems. This is striking because memory difficulties appear to be the most frequent cognitive symptom in subjects with endocrine dysfunction, possibly because the hippocampus is a major target of GC and other hormones. On the other hand, it should be remarked that experiments in rodents have revealed that high GC levels disrupt executive components of memory by inducing atrophy in prefrontal areas [46]. In humans, a similar effect has been reported, but only for subjects with mild cognitive impairment (MCI), not for healthy elderly and subjects with Alzheimer disease [47].

Lastly, establishment of sequential/temporal cause-effect relationships between TBI, hypopituitarism (including the role of individual hormones) and behavioral impairments must await the development of suitable paradigms in animals; such studies will also facilitate deeper exploration of the cellular/neuroplastic mechanisms that underlie the hormone-mediated effects of TBI on mental functions.

## 4. Growth Hormone (GH) Replacement for Mental Dysfunction after TBI

In light of the strong evidence suggesting that TBI-induced impairments of mental function are causally related to injury-associated reductions in hormone secretion, it is reasonable to expect that hormone replacement therapy would partially or fully restore mental functions or, at the very least, facilitate post-injury rehabilitation. Indeed, promising results have been reported on the effects of hormone replacement in growth hormone-deficient subjects. For example, a pilot study by Maric *et al.* [48] found significantly improved verbal and visual memory and significantly reduced depressive and anxiety symptoms in six TBI patients with GH deficiency following six months of GH therapy. In a randomized placebo-controlled study of 23 TBI subjects with GH deficiency, High *et al.* [49] found significant improvements in overall mental speed after one year of GH replacement; however, the authors failed to observe treatment-associated changes in specific cognitive abilities such as memory and executive function. Similarly, Reimunde *et al.* [50] found a general improvement in cognition in a small placebo-controlled study (*n* = 11) on TBI subjects that had undergone GH therapy. Lastly, a randomized control study (23 GH-treated and 27 placebo-treated subjects that had suffered TBI > 5 years previously) found modest, but significant, improvements in overall mental speed and verbal memory effects of GH replacement [51].

GH therapy has proven to be effective in improving cognition and mood in other patient groups with hypopituitarism (e.g., [52,53]). However, the preliminary evidence of beneficial treatment effects of GH replacement for cognitive deficits in subjects with TBI is too weak to draw conclusions about efficacy and importantly, specificity. Although the literature reports improvements in a variety of cognitive domains (especially in mental speed), this does not necessarily exclude effects on specific domains (e.g., executive function and memory). Since GH deficiency represents probably the most common pituitary disorder after TBI [54], research on GH replacement in TBI subjects deserves priority. Nevertheless, it would be important to examine also the potential benefits of hormone replacement for TBI patients displaying concomitant impairments of mental and adrenal, and/or thyroid function.

## 5. Conclusions and Recommendations

It becomes clear from the experimental and clinical data available, that hormones play an important role in maintaining optimal brain functioning in the adult. Subjects with endocrine dysfunction may exhibit mental disorders, spanning the cognitive, affective and/or behavioral domains. Notably, subjects with endocrine alterations show more global, unspecific cognitive dysfunction, with mental slowing and impaired executive regulatory process being dominant features; these can be alleviated by GH replacement. This observation fits with the idea of a final common pathway disorder, which is based on the premise that any type of dysfunction of the complex network underlying executive, regulatory components of cognition and mood may produce a similar pattern of disorders [55,56]. While more studies are needed to strengthen this view, support comes from the fact that frontal brain structures are relatively well endowed with hormone receptors [57].

Subjects with TBI frequently present with cognitive and affective disorders, but disentangling the causes of these (morphological injury *vs.* primary neuroendocrine dysfunction) remains a challenge. The evidence briefly reviewed here underscores the need for the identification and timely appropriate clinical management of hormone deficiencies, as well as for a comprehensive neuropsychological and psychopathological assessment in subjects with TBI. Likewise, the need for endocrine profiling and replacement in subjects with hypopituitarism deserves further attention, including the definition of criteria regarding indication of hormone replacement and evaluation of treatment efficacy (see [58]). The latter will require comprehensive cognitive and affective assessments of individuals using standardized and robust neuropsychological tests ([3,59]; *cf.* Table 1 and Table 2). Together, these considerations will help develop appropriate strategies for therapeutic measures involving neuropsychological rehabilitation for TBI patients.

**Table 1:** Mental domains and functional significance.

Domains	Functional Significance	
**Attention**	**	
Alertness (vigilance)	preparedness to respond or act	
Information processing capacity	speed and accuracy of information processing	
Sustained attention	maintenance of attention at a given level for an extended period of time	
Divided attention	simultaneous attention to two or more stimuli/actions (prerequisite to perform concurrently two or more tasks)	
Spatial attention	distribution of attention in space (global (parallel) processing, local processing)	
**Memory**		
Short-term memory	recall (reproduction or recognition) of a limited number of stimuli without further elaboration	
Working memory	recall (reproduction or recognition) of a limited number of stimuli with further elaboration	
Episodic memory	memory for specific, personally experienced events in a given context (time, place, *etc*.)	
Semantic memory	memory for general or domain-specific knowledge or information	
Verbal memory	capacity to remember (reproduction or recognition) written or spoken material	
Visual memory	capacity to remember (reproduction or recognition) visual images	
**Executive function**		
Planning	mental outline of the steps required to perform a task or solve a problem	
Problem solving	process by which individuals attempt to solve a complex task	
Cognitive flexibility	objective appraisal and appropriate flexible action, e.g., ability to change between visual or verbal stimuli or between actions	
Multi-tasking	ability to perform more than one action/task at a time, requiring multiple information processing, working memory, divided attention, and monitoring of actions	
**Affective state/mood**	any type of emotional state, associated with an emotional response bias for days or weeks	

**Table 2:** Recommendations for a standardized assessment of cognition and affective state/mood (for a detailed and comprehensive description of assessment measures, see [60]).

Domains	Test(s)
**Premorbid intelligence**	Verbal IQ-scale of the WAIS-R; National Adult Reading Test (NART)
**Attention**	
Alertness (vigilance)	Reaction time with (phasic alertness) and without warning signals (tonic alertness)
Information processing capacity	Trail Making Test (TMT) A
Sustained attention	Continued Performance Tests (CPT)
Divided attention Test (PASAT)	Stroop Test; Symbol Digit Modalities Test (SDMT); Paced Auditory Serial Addition
Spatial attention	cancellation tests; visual search tests
**Memory**	
Short-term memory	digit span forward; block tapping forward
Working memory	digit span backward; block tapping backward
Semantic memory	
Verbal memory	Auditory Verbal Learning tests; story recall (Logical Memory; WMS III/IV)
Visual memory	Visual reproductions (WMS III/IV); Camden Memory tests
**Executive function**	**
Planning/problem solving	Raven’s Coloured Progressive Matrices (RCPM); Porteus Maze Test; Tower of London Test
Cognitive flexibility	Trail making Test (TMT) B; Wisconsin Card Sorting Test (WCST); verbal fluency tests (COWAT)
Multi-tasking	Symbol Digit Modalities Test (SDMT); Paced Auditory Serial Addition Test (PASAT); Stroop Tests
**Affective state/mood**	Hospital anxiety and depression scale (HADS); Geriatric depression scale (GDS)

Given the multifactorial nature of TBI, it is imperative that an interdisciplinary approach is developed to improve clinical outcomes [59,60]. However, management and prognosis for patients with TBI-induced hypopituitarism are confounded by “the marked variability in type, location and degree of pathological changes following a TBI, as well as the equally ubiquitous variability in pre-morbid organic and psychological functioning …” [60]. This explains the present equivocal conclusions regarding the impact of TBI-related hormone deficiency on mental function and chronic complaints of fatigue [61,62,63].

## Supplementary Files

Supplementary File 1
