# Supplementary material for: Neuropsychology of Neuroendocrine Dysregulation after Traumatic Brain Injury
Source: J Clin Med. 2015 May 20;4(5):1051–62. doi: 10.3390/jcm4051051 (PMC4470216; doi:10.3390/jcm4051051)
Supplement: Supplementary File 1 [file jcm-04-01051-s001.pdf]

Supplementary Information

Table S1. Mental functions and brain structures involved (simplified synopsis).

Domains	Brain Structures
**Perception**	
Vision	occipital lobe (striate and extrastriate visual areas)
Audition	temporal lobe (primary and secondary auditory areas)
Body perception	parietal lobe (somatosensory areas)
Gustation	thalamus, hypothalamus, temporal and orbitofrontal areas
Olfaction	thalamus, temporal and orbitofrontal areas
**Attention**	
Alertness (vigilance)	reticular formation (brain stem, midbrain)
Selective attention	thalamus, prefrontal cortex
Divided attention	prefrontal cortex
Spatial attention	posterior parietal cortex; thalamus
Attention regulation (executive control)	prefrontal cortex
**Memory**	
Episodic memory	hippocampus, thalamus, temporal and prefrontal areas
Semantic memory	hippocampus, (temporal) association areas
Procedural memory	premotor areas, basal ganglia, cerebellum
**Executive functions**	(lateral) prefrontal areas
**Affective regulation**	orbitofrontal areas, limbic system (incl. temporal lobe), insula

Table S2. Mental domains and functional significance.

Domains	Functional Significance
**Attention**	
Alertness (vigilance)	preparedness to respond or act
Information processing capacity	speed and accuracy of information processing
Sustained attention	maintenance of attention at a given level for an extended length of time
Divided attention	attention to two or more stimuli/actions at the same (prerequisite to perform concurrently two or more tasks)
Spatial attention	distribution of attention in space (global (parallel) processing, local processing)
**Memory**	
Short-term memory	recall (reproduction or recognition) of a limited number of stimuli without further elaboration
Working memory	recall (reproduction or recognition) of a limited number of stimuli with further elaboration
Episodic memory	memory for specific, personally experienced events in a given context (time, place, *etc.*)
Semantic memory	memory for general or domain-specific knowledge or information
Verbal memory	capacity to remember (reproduction or recognition) written or spoken material
Visual memory	capacity to remember (reproduction or recognition) visual images

Table 2. *Cont.*

Domains	Functional Significance
**Executive function**	
Planning	mental outline of the steps required to perform a task or solve a problem
Problem solving	process by which individuals attempt to overcome difficulties in a given complex task condition
Cognitive flexibility	objective appraisal and appropriate flexible action, e.g., ability to change between visual or verbal stimuli or between actions
Multi-tasking	ability to perform more than one action/task at a time, requiring multiple information processing, working memory, divided attention, and monitoring of actions
**Affective state/mood**	any type of emotional state, associated with an emotional response bias for days or weeks

Table S3. Recommendations for a standardized assessment of cognition and mood (for a detailed and comprehensive description of assessment measures, see [1]).

Domains	Test(s)
**Premorbid intelligence**	Verbal IQ-scale of the WAIS-R; National Adult Reading Test (NART)
**Attention**	
Alertness (vigilance)	Reaction time with (phasic alertness) and without warning signals (tonic alertness)
Information processing capacity	Trail Making Test (TMT) A
Sustained attention	Continued Performance Tests (CPT)
Divided attention	Stroop Test; Symbol Digit Modalities Test (SDMT); Paced Auditory Serial Addition Test (PASAT)
Spatial attention	cancellation tests; visual search tests
**Memory**	
Short-term memory	digit span forward; block tapping forward
Working memory	digit span backward; block tapping backward
Semantic memory	
Verbal memory	Auditory Verbal Learning tests; story recall (Logical Memory; WMS III/IV)
Visual memory	Visual reproductions (WMS III/IV); Camden Memory tests
**Executive function**	
Planning	Tower of London Test; Porteus Maze Test
Problem solving	Tower of London Test; Raven's Coloured Progressive Matrices (RCPM);
Cognitive flexibility	Trail making Test (TMT) B; Wisconsin Card Sorting Test (WCST); verbal fluency tests (COWAT)
Multi-tasking	Symbol Digit Modalities Test (SDMT); Paced Auditory Serial Addition Test (PASAT); Stroop Tests
**Affective state/mood**	Beck Depression Inventory (BDI); Hospital Anxiety and Depression Scale (HADS); Geriatric Depression Scale (GDS)

Reference

1. Behan, L.A.; Phillips, J.; Thompson, C.J.; Agha, A. Neuroendocrine disorders after traumatic brain injury. *J. Neurol. Neurosurg. Psychiatry* **2008**, *79*, 753–759.
